# Structural reliability calculation method based on the dual neural network and direct integration method

**DOI:** 10.1007/s00521-016-2554-7

**Published:** 2016-08-23

**Authors:** Haibin Li, Yun He, Xiaobo Nie

**Affiliations:** College of Science, Inner Mongolia University of Technology, Hohhot, China

**Keywords:** Reliability, Dual neural network, Direct integral method, Rational neural network

## Abstract

Structural reliability analysis under uncertainty is paid wide attention by engineers and scholars due to reflecting the structural characteristics and the bearing actual situation. The direct integration method, started from the definition of reliability theory, is easy to be understood, but there are still mathematics difficulties in the calculation of multiple integrals. Therefore, a dual neural network method is proposed for calculating multiple integrals in this paper. Dual neural network consists of two neural networks. The neural network A is used to learn the integrand function, and the neural network B is used to simulate the original function. According to the derivative relationships between the network output and the network input, the neural network B is derived from the neural network A. On this basis, the performance function of normalization is employed in the proposed method to overcome the difficulty of multiple integrations and to improve the accuracy for reliability calculations. The comparisons between the proposed method and Monte Carlo simulation method, Hasofer–Lind method, the mean value first-order second moment method have demonstrated that the proposed method is an efficient and accurate reliability method for structural reliability problems.

## Introduction

From the middle of the last century, the engineering problems with random variables have been paid many attentions. Up to now, many methods for structural reliability problems have been developed. These methods can be divided into three parts. One is a reliability analysis method to solve the response of the structure or probability characteristic of function, such as the first-order second moment method (FOSM) [1] and high-order moment method [2, 3]. The estimated probability forms of function are used to calculate structural reliability based on each moment. The error will be large under the difference between actual probability form and ideal probability form. Although the accuracy can be improved by finding out higher-order moments, the computational burden is improved. Furthermore, the accuracy of these methods is lower for highly nonlinear function problems [4]. Other widely used methods are direct sampling Monte Carlo reliability calculation methods. The large number of samples is generally required for these methods to perform reliability analysis with high precision. In order to reduce the computational burden, several improved algorithms have been developed such as importance sampling method [5], stratified sampling method [6], Markov chain sampling method [7]. However, the application for large and complex structures is still time-consuming.

The third widely used method is direct integral method [8]. The integral equation of reliability is calculated directly according to the definition of reliability. The reliability can be calculated based on the multiple integrals of the joint probability density functions on safe domain of the system. The direct integral method includes three aspects of technology, that is, the rules of the integral area, the constitution of the probability density function and the multiple integral [9]. The rules of the integral area had been successfully solved through the introduction of performance function. The calculation of multiple integral is research emphasis on this kind of method. Numerical integration problems of three and lower dimensions efficiently solved by Gaussian integral formula. The problems of multiple integral are generally divided into two categories: certainty and uncertainty algorithm. Uncertainty algorithm refers to Monte Carlo method and it is improved. This method is the same as Monte Carlo method in above. Certainty algorithm has Toeplitz matrix method [10], Lagrange function method [11], two-sided estimates method [12], discrete Adomian decomposition method [13], positive definite function method [14], triangular functions (TF) method [15] and other quadrature formula methods. There are also Markov chain splitting (MCS) method [16], generalized Mellin–Barnes method [17], log gamma function method [18] and other new methods. Through the above methods, we can obtain approximate analytical solution and numerical solution of various multidimensional integral, but these methods have their limitation. For example, in quadrature formula method, integral equation can be discretized an algebraic system. However, solving its massive algebraic system is also a thorny problem if its accuracy is guaranteed. MCS method has extensive range to calculate reliability, but there are the same shortcomings as the quadrature formula method. Generalized Mellin–Barnes method and log gamma function method can calculate simple and specific multidimensional integral, but its accuracy cannot be guaranteed for more complex multidimensional integral. With the deepening research on certainty algorithm, the direct integral method can play bigger role in structural reliability calculation.

In conclusion, three kinds of structural reliability calculation have following problems. (1) Based on solving structural response or moments of performance function, several methods are not adapt to the problem of multiple failure models and its computational accuracy seriously depends on the form of performance function. (2) Monte Carlo method has huge computation for structural reliability calculating. (3) In the type of certainty direct integral, reliability calculation method encounters mathematical difficulties in the multiple integral and quantitative description of the computational accuracy.

Artificial neural network is an adaptive nonlinear dynamic system. It composes a large number of simple neurons that connect each other. Artificial neural network acts as a result of biocybernetics. Its tentacles almost has extended to various engineering fields. Goodacre et al. [19] used neural network to achieve the multivariate data analysis. Freitas and Prat [20] studied phase distribution of the liquid surface tension gradient with network model. Bayesian hybrid modeling method was proposed with neural network in Ref. [21]. Kocadagli [22] put forward a novel hybrid learning algorithm for full Bayesian of artificial neural network. It has wide application. The main characteristics of general neural network are as follows. It has strong self-learning and self-adaptive ability and better effect in the nonlinear mapping processing. But it has some shortcomings, such as strong dependence of sample, slow convergence speed and it easily enters the local minimum value.

Neural network has the capability of approximating any functions, so it can be applied to structural reliability properly. Lopes [23] used neural network instead of finite element analysis to reliability analysis, and the neural network had advantages in computational efficiency when compared to MCS. Cheng [24] used the neural network to construct the limit-state function of long span bridge, genetic algorithm (GA) is employed to train the network, and the failure probability of structure was received. Zou et al. [25] used neural network to fit the performance function of structure, the values of performance function and partial derivatives at the point of mean values are got, and the moments of performance function are calculated based on the moments of random variables.

Neural network has problems in the calculation of reliability. The one is that the approximate function constructed using neural network by using the same training sample is uncertain due to the randomness of weights and threshold value of the neural network. The relationship between the excitation and response outside the sample points is difficult to be effectively guaranteed. In order to improve the accuracy, the number of samples can be increased. In practical engineering, total samples for training are limited; therefore, calculating accuracy based on neural network cannot be guaranteed effectively. Another is that the training neural network surface could go through sample points exactly, but the derivative value in the sample points does not meet the requirements. Therefore, the higher-order moment method based on the derivation cannot meet the requirements. According to the multiple integral problems, Ref. [26] provided a direct integral method based on the triangle basis neural network. However, the computational efficiency is low because many triangle basis functions are required to guarantee the accuracy. Some scholars used neural network to calculate multidimensional integral, such as Volterra integral [27], Choquet integral [28, 29], stochastic integral [30], fuzzy integral [31]. The main idea is that the integral problem is equaled to mapping problem between integrand and original function. But lacking of sample and low generalization ability restrict to improve accuracy.

In this paper, an integration method is proposed based on dual neural network, which can be applied for reliability calculation directly. The proposed method constructs two same neural networks structure, which have multiple inputs, single output and single hidden. Through designing function relation between the weights of two neural networks, it makes one neural network approximate integrand and the other neural network approximate original function. Therefore, the above networks are, respectively, called integrand neural network and original function neural network. We only need to train integrand neural network. Thus, the weights of original function neural network are given directly by function relation between the weights of two neural networks. Then we use the original function neural network to calculate multiple integral. With proposed method, integrand can easily obtain sample data that are directly trained; the integral computational accuracy will be greatly improved. So the proposed method is an efficient and accurate reliability method for structural reliability problems. This paper is organized as follows. Firstly, it introduces the integral form of the reliability computation and the normalization method of the integral area; secondly, this paper gives the key content, which is a kind of dual integral method based on neural network, and then is the simulation part, to verify the effectiveness of the proposed method; Finally, the full-text conclusion and the prospect for the future are given.

## The integral form of structural reliability calculation

Structural reliability can be expressed as a probability of a function *Y* > 0. *Y* can be expressed as a function of the basic variables *X* = [*x*_1_, *x*_2_, …, *x*_*n*_], that is, *Y* = *g*(*x*_1_, *x*_2_, …, *x*_*n*_). Let *X* is a random vector and the probability density function of the function is *F*(*Y*), and structure reliability can be written as

1 $$P = \int_{Y > 0} {F(Y){\text{d}}Y}$$

Generally, *F*(*Y*) cannot be determined accurately; therefore, Eq. (1) cannot be used to calculate system reliability directly. To solve the problem, the principle of the probability conservation is considered. According to the principle of the probability conservation, probability of any d*x* in *X* equals to probability of corresponding d*y* in mapping *Y*, and the probability conservation can be expressed as Eq. (2)

2 $$F(Y){\text{d}}Y = H(x_{1} ,x_{2} , \ldots ,x_{n} ){\text{d}}x_{1} {\text{d}}x_{2} \ldots {\text{d}}x_{n}$$

where *H*(*x*_1_, *x*_2_, …, *x*_*n*_) is the joint probability density function of the input variables. According to Eqs. (1) and (2), Eq. (1) can be obtained as

3 $$P = \int_{{}}^{{}} \cdots \int_{{g(x_{1} ,x_{2} , \ldots ,x_{n} ) > 0}}^{{}} {H(x_{1} ,x_{2} , \ldots ,x_{n} ){\text{d}}x_{1} {\text{d}}x_{2} \ldots {\text{d}}x_{n} }$$

There are two difficulties in solving Eq. (3) directly. Firstly, it is difficult to get the original function. Secondly, the boundary condition is very complicated. Solve the problem of boundary complexity. Introduce a weight function $$h(x) = \left\{ {\begin{array}{*{20}l} {1,} \hfill & {x > 0} \hfill \\ {0,} \hfill & {x \le 0} \hfill \\ \end{array} } \right.$$

Let $$\int_{{}}^{{}} \cdots \int_{{g(x_{1} ,x_{2} , \ldots ,x_{n} ) \le 0}}^{{}} {h(g(x_{1} ,x_{2} , \ldots ,x_{n} ))H(x_{1} ,x_{2} , \ldots ,x_{n} ){\text{d}}x_{1} {\text{d}}x_{2} \ldots {\text{d}}x_{n} = 0}$$

Then Eq. (3) becomes

4 $$\begin{aligned} P & = \int_{{}}^{{}} \cdots \int_{{{{g(x}}_{ 1} , {\text{x}}_{ 2} , {{ \ldots ,x}}_{{n}} ) { > 0}}}^{{}} {{{h(g(x}}_{ 1} , {{x}}_{ 2} , {{ \ldots ,x}}_{{n}} ) ) {{H(x}}_{ 1} , {{x}}_{ 2} , {{ \ldots ,x}}_{{n}} ) {\text{d}}x_{ 1} {\text{d}}x_{2} \ldots {\text{d}}x_{n} } \\ & \quad + \int_{{}}^{{}} \cdots \int_{{{{g(x}}_{ 1} , {{x}}_{ 2} , {{ \ldots ,x}}_{{n}} ) {{\leq0}}}}^{{}} {{{h(g(x}}_{ 1} , {{x}}_{ 2} , {{ \ldots ,x}}_{{n}} ) ) {{H(x}}_{ 1} , {{x}}_{ 2} , {{ \ldots ,x}}_{{n}} ) {\text{d}}x_{ 1} {\text{d}}x_{2} \ldots {\text{d}}x_{n} } \\ & = \int_{ - \infty }^{\infty } \cdots \int_{ - \infty }^{\infty } {h(g(x_{1} ,x_{2} , \ldots ,x_{n} ))H(x_{1} ,x_{2} , \ldots ,x_{n} ){\text{d}}x_{1} {\text{d}}x_{2} \ldots {\text{d}}x_{n} } \\ \end{aligned}$$

where *g*(*x*_1_, *x*_2_, …, *x*_*n*_) is the performance function.

Let

5 $$y\left( {x_{1} , x_{2} , \ldots , x_{n} } \right) = h\left( {g\left( {x_{1} , x_{2} , \ldots , x_{n} } \right)} \right)H\left( {x_{1} , x_{2} , \ldots , x_{n} } \right)$$

Equation (4) can be rewritten as

6 $$P = \int_{{\mu_{n} - n\sigma_{n} }}^{{\mu_{n} + n\sigma_{n} }} \cdots \int_{{\mu - n\sigma_{1} }}^{{\mu + n\sigma_{1} }} {y(x_{1} ,x_{2} , \ldots ,x_{n} ){\text{d}}x_{1} {\text{d}}x_{2} \cdots {\text{d}}x_{n} }$$

In Eq. (6), *μ*_*i*_ and *σ*_*i*_ are, respectively, the mean and standardized deviation of random variable *i*. *n* is a positive integer, and the value of n usually *n* ≥ 3 which is determined by accuracy requirements.

Let the original function of the integrand *y*(*x*_1_, *x*_2_, …, *x*_*n*_) in Eq. (6) is *Y*(*x*_1_, *x*_2_, …, *x*_*n*_), according to multiple integral theory, and the function *Y* is the weighted algebraic sum of each vertex in a hypercube *D* = {(*x*_1_, *x*_2_, …, *x*_*n*_)|*x*_1_^1^ ≤ *x*_1_ ≤ *x*_1_^2^, *x*_2_^1^ ≤ *x*_2_ ≤ *x*_2_^2^, …, *x*_*n*_^1^ ≤ *x*_*n*_ ≤ *x*_*n*_^2^}, which can be expressed as

7 $$J = \sum\limits_{{m_{1} = 1}}^{2} {\sum\limits_{{m_{2} = 1}}^{2} { \cdots \sum\limits_{{m_{n} = 1}}^{2} {( - 1)^{{\sum\limits_{k = 1}^{n} {m_{k} } }} Y(x_{1}^{{m_{1} }} ,x_{2}^{{m_{2} }} , \ldots ,x_{n}^{{m_{n} }} )} } }$$

In this paper, the dual neural network will be used to solve the original function of Eq. (6).

## Multiple integral calculation method based on the dual neural network

**Fig. 1:**
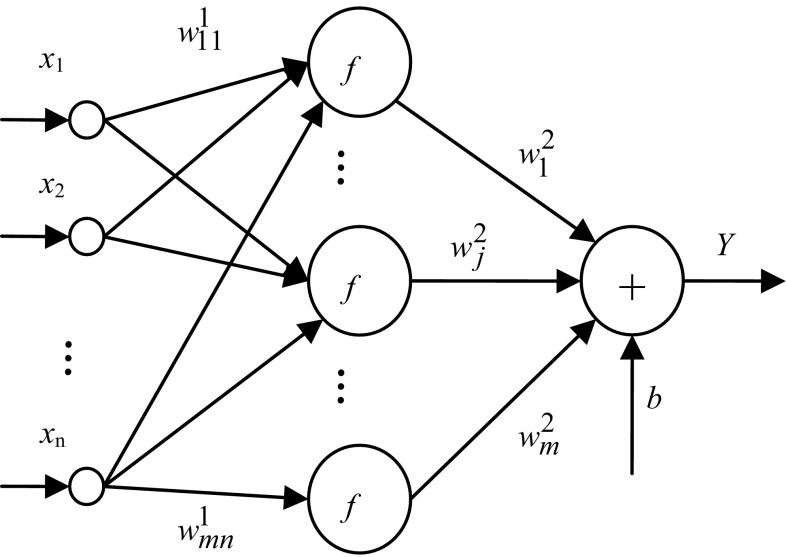
Structure of original function network B

Based on principle of neural network BP, network B is used to establish the relationship of input variables *X* to the original function *Y*. The network structure is shown in Fig. 1.

The relationships between the output and input can be expressed as

8 $$Y = \sum\limits_{j = 1}^{m} {f\left( {\sum\limits_{i = 1}^{n} {w_{ji}^{1} x_{i} + \vartheta_{j} } } \right)w_{j}^{2} + b}$$

The derivation of Eq. (8) can be given by

9 $$y = \frac{{\partial^{n} Y}}{{\partial x_{1} \partial x_{2} \ldots \partial x_{n} }} = \sum\limits_{j = 1}^{m} {f^{(n)} \left( {\sum\limits_{i = 1}^{n} {w_{ji}^{1} x_{i} + \vartheta_{j} } } \right)w_{j1}^{1} w_{j2}^{1} \ldots w_{jn}^{1} w_{j}^{2} }$$

where *W*_*j*_ = *w*_*j*1_^1^ *w*_*j*2_^1^…*w*_*jn*_^1^ *w*_*j*1_^2^,

Equation (9) can be rewritten as a function of the relationship between the output and input variables as below.

10 $$y = \sum\limits_{j = 1}^{m} {f^{(n)} \left( {\sum\limits_{i = 1}^{n} {w_{ji}^{1} x_{i} + \vartheta_{j} } } \right)W_{j} }$$

**Fig. 2:**
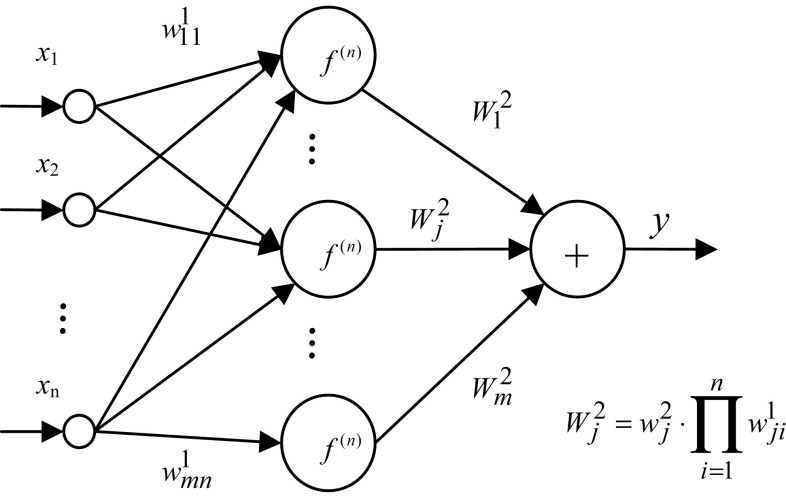
Structure of integrand network A

The network, mapping relationship between the input variable *X* to the function *y*, is called neural network A. The structure of the network A is shown in Fig. 2.

Obviously, the original function of the integrand function *y* in the multiple integral Eq. (6) is *Y*. Accordingly, a dual neural network is composed of neural network A and neural network B. The neural networks A and B are, respectively, called the integrand function network and original function network.

From Eqs. (8) and (10), two networks with three layers, namely integrand function network and the original function of network, have *n* inputs, single output and *m* unit in hidden layer. Let the connection weights and threshold of input layer to hidden layer unit are, respectively, *w*_*ji*_^1^ and *ϑ*_*j*_, the connection weights and threshold of hidden layer to output layer are *W*_*j*_ and 0, and the activation function of the hidden layer units is *f* ^(*n*)^; then, in the integrand network, the connection weights and threshold of input layer to hidden layer unit are *w*_*ji*_^1^ and *ϑ*_*j*_, the connection weights and threshold of hidden layer to output layer are *W*_*j*_/∏_*i*=1_^*n*^ *w*_*ji*_^1^ and *b*, and the activation function of the hidden layer units is *f*. When the network A approximates to the integrand, the network B approximates the original function.

In problems, set activation function of hidden layer units is *f* = *e* ^*x*^ in network B. At this moment, the activation function of hidden layer units is *f* ^(*n*)^ = *e* ^*x*^ in network A. These two neural networks have the same activation function. At the same time, Ref. [32] has verified that the activation function of hidden layer units is exponential function that has better accuracy and generalization ability than the traditional logsig, tansig activation function. Because learning rate of stability requirement is low, the gradient descent method makes training slow. Although the speed of the momentum gradient method is improved, it still cannot meet the requirements of practical application. Convergence speed of Newton’s method is quick. But in each process of iteration, we need to calculate second derivative of error function—Hessian matrix that makes computational quantity very large. If the error performance function in neural network is the form of sum of squares, we can use Levenberg–Marquardt training algorithm (LM algorithm). This algorithm does not need to calculate the Hessian matrix; Hessian matrix can be approximately substituted by following matrix. *H* = *J* ^*T*^ *J*. Where the *J* is jacobian matrix, it is about the function of weights and thresholds; the training algorithm can be written as Eq. (11).

11 $$w_{k + 1} = w_{k} - [J^{T} J + \mu I]^{ - 1} J^{T} e$$

where *w*_*k*_ is the corresponding weights vector in neural network when *k*th training step, *e* is the error vector of network, *μ* is dynamic adjustable parameter. When *μ* = 0, the LM algorithm is equal to approximate Newton’s method that has Hessian matrix. When *μ* is big, it is equal to gradient method that have short step. In the training process of iteration, if success, it will decrease the value of *μ*; if failure, it will increase the value of *μ*. Thus, the performance function will decrease more and more. When network A trains, the proposed method adopts the Levenberg–Marquardt training algorithm based on the above theory.

## Numerical example

### Example 1

Assume that the performance function of a system is *g*(*x*_1_, *x*_2_) = *x*_1_^3^ + *x*_2_^3^ − 18, where *x*_1_ and *x*_2_ are independent normally distributed random variables with the mean value *μ*_*x*1_ = *μ*_*x*2_ = 10 and standard deviation *σ*_*x*1_ = *σ*_*x*2_ = 5, respectively. Mathematically, the joint probability density function of the input variables can be written as:

$$H(x_{1} ,x_{2} ) = \frac{1}{{2\pi \sigma_{1} \sigma_{2} }}\exp \left\{ { - \frac{1}{2}\left[ {\frac{{(x_{1} - \mu_{1} )^{2} }}{{\sigma_{1}^{2} }} + \frac{{(x_{2} - \mu_{2} )^{2} }}{{\sigma_{2}^{2} }}} \right]} \right\}$$

*H*(*x*_1_, *x*_2_) and *g*(*x*_1_, *x*_2_) are substituted for Eq. (5) to obtain the integrand. The range of *x*_1_ and *x*_2_[*μ*_*x*1_ − 4*σ*_*x*1_, *μ*_*x*1_ + 4*σ*_*x*1_] and [*μ*_*x*2_ − 4*σ*_*x*2_, *μ*_*x*2_ + 4*σ*_*x*2_] are, respectively, divided into 100 parts. Two variables cross each other to form network input samples. The integrand function is used to calculate output values the corresponding input sample points. Network A training sample set is shown in Table 1.

**Table 1:** Training sample set of network A in example 1

*x*_2_	*x*_1_					
	*y*					
	−10	−9.6	−9.2	…	29.6	30
−10	0	0	0	…	0.0983e−8	0.0716e−8
−9.6	0	0	0	…	0.1350e−8	0.0983e−8
−9.2	0	0	0	…	0.1841e−8	0.1341e−8
…	…	…	…	…	…	…
29.6	0.0983e−8	0.1350e−8	0.1841e−8	…	0.1350e−8	0.0983e−8
30	0.0716e−8	0.0983e−8	0.1341e−8	…	0.0983e−8	0.0716e−8

**Fig. 3:**
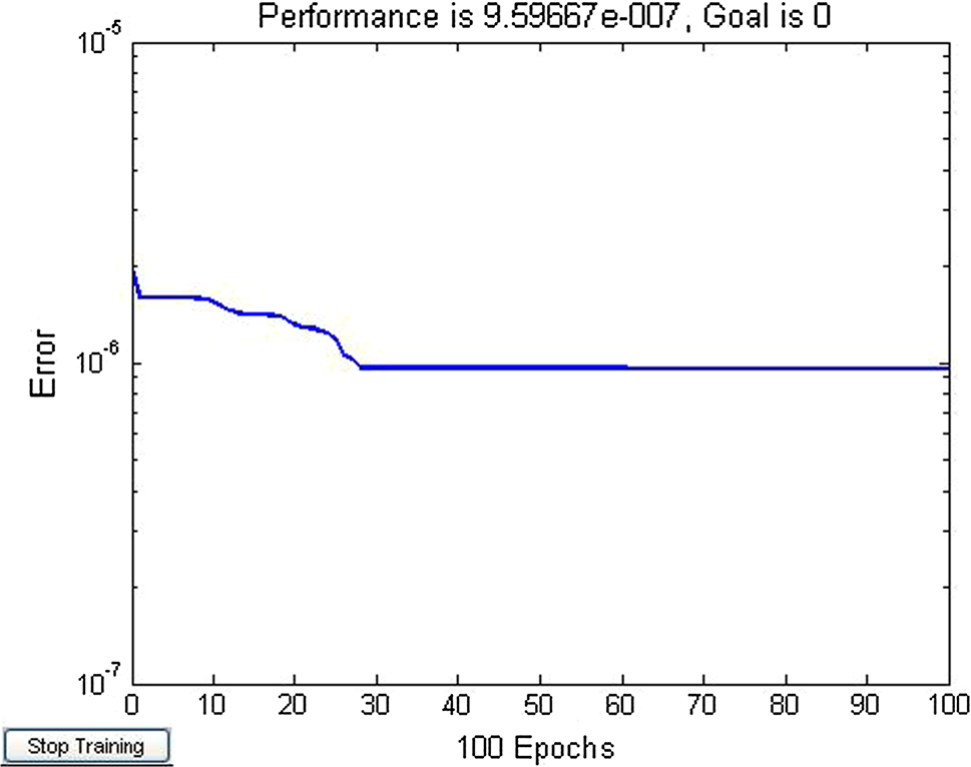
Training error curve of network A in example 1

Activation function of hidden layer units is *f* = *e* ^*x*^. This example use Levenberg–Marquardt training algorithm. Set neurons number of neural network A, B is *s* in hidden layer. After adjustment, the hidden layer neurons *s* = 15. Training integrand network A 100 steps, error convergence curve is shown in Fig. 3.

The relationship of the dual neural network is used to construct original function network B. Network A training sample set of each vertex in a hypercube is calculated and simulated with network B; the sample set is shown in Table 2. The results are substituted to Eq. (7) to get the result of reliability.

**Table 2:** Simulative sample set of network B in example 1

*x*_1_	30	30	−10	−10
*x*_2_	30	−10	30	−10

In order for accuracy comparisons, the results calculated using the mean value first-order second moment (MVFOSM) method, Hasofer–Lind (HL) method and Monte Carlo simulation (MCS) method are also given. The result calculated using MCS with sampling 1,000,000 is viewed as accurate solutions. The relative errors compared with MCS are shown in Table 3.

**Table 3:** Results calculated using different method

Methods	The proposed method	MVFOSM	HL	MCS
Reliability	0.9966	0.8249	0.9875	0.9945
Relative error (%)	0.21	17.05	0.7	0

From Table 3, it can be seen that the accuracy of the proposed method is higher than MVFOSM and HL when compared to MCS. The accuracy of the MVFOSM and HL are very low because the performance function is a highly nonlinear function. The efficiency of the proposed method is higher than MCS significantly since the only 100 steps are used for the proposed methods when compared to 1,000,000 trials for MCS.

### Example 2

**Fig. 4:**
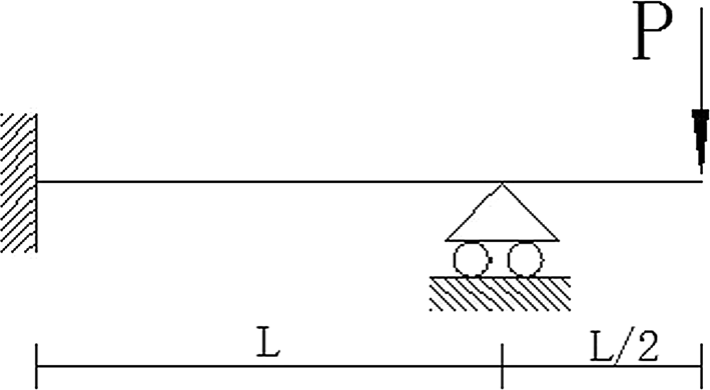
Plane frame structure

A plane frame structure is shown in Fig. 4; the performance function of displacement is expressed as: $g(P,E,I) = \frac{{5PL^{3} }}{48EI} - \frac{L}{30}$, where *P* is concentrated load; *L* is the length of the beam and $L = 5\;{\text{m}}$; *E* is the modulus of elasticity; *I* is the inertia moment of the cross section. *P*, *E* and *I* are random variables. *E* and *I* are normally distributed; *P* is extreme value distribution of type I. The means *μ*_*P*_, *μ*_*E*_, *μ*_*I*_ of *P*, *E*, *I* are, respectively, 4 kN, 2.0 × 10^7^ kN/m^2^, 1 × 10^−4^ m^4^.

According to the above example, the joint probability density function of input variables *P*, *E*, *I* can be given by

$$\begin{aligned} H(P,E,I) & = \alpha \exp \{ - (P - \delta )\alpha - \exp [ - (P - \delta )\alpha ]\} \\ & \quad \times \frac{1}{{2\pi \sigma_{E} \sigma_{I} }}\exp \left\{ { - \frac{1}{2}\left[ {\frac{{(E - \mu_{E} )^{2} }}{{\sigma_{E}^{2} }} + \frac{{(I - \mu_{I} )^{2} }}{{\sigma_{I}^{2} }}} \right]} \right\} \\ \end{aligned}$$

where *δ* = 3.5499, *α* = 1.2825.

*H*(*P*, *E*, *I*) and *g*(*P*, *E*, *I*) are substituted into Eq. (5); then, integrand can be obtained.

Variables *P*, *E*, *I* are, respectively, divided into 10 parts in the range of [*μ*_*P*_ − 4*σ*_*P*_, *μ*_*P*_ + 4*σ*_*P*_], [*μ*_*E*_ − 4*σ*_*E*_, *μ*_*E*_ + 4*σ*_*E*_] and [*μ*_*I*_ − 4*σ*_*I*_, *μ*_*I*_ + 4*σ*_*I*_]. Each of the two cross-variables is used as input samples for the network, and the integrand function is employed to the corresponding sample point of the network output value. In order to avoid *g*(*P*, *E*, *I*) is zero, the first point of *E* is −0.2 that instead of a smaller value. Network A training sample set is shown in Table 4.

**Table 4:** Training sample set of network A in example 2

*E, I* (10^7^kN/m^2^, 10^−4^ m^4^)	*y*						
	*P* (kN)	0	0.8	1.6	…	7.2	8.0
−0.2 e−7	0.2	0	0	0	…	0.0021e−6	0.0008e−6
−0.2 e−7	0.36	0	0	0.0003e−6	…	0.0376e−8	0.0135e−6
−0.2 e−7	0.52	0	0	0.0024e−6	…	0.3529e−6	0.1273e−6
…	…	…	…	…	…	…	…
−0.2 e−7	1.64	0	0	0.0003e−6	…	0.0376e−6	0.0135e−6
−0.2 e−7	1.8	0	0	0	…	0.0021e−6	0.0008e−6
…	…	…	…	…	…	…	…
4	1.64	0	0	0.0003e−6	…	0.0376e−6	0.0135e−6
4	1.8	0	0	0	…	0.0021e−6	0.0008e−6

**Fig. 5:**
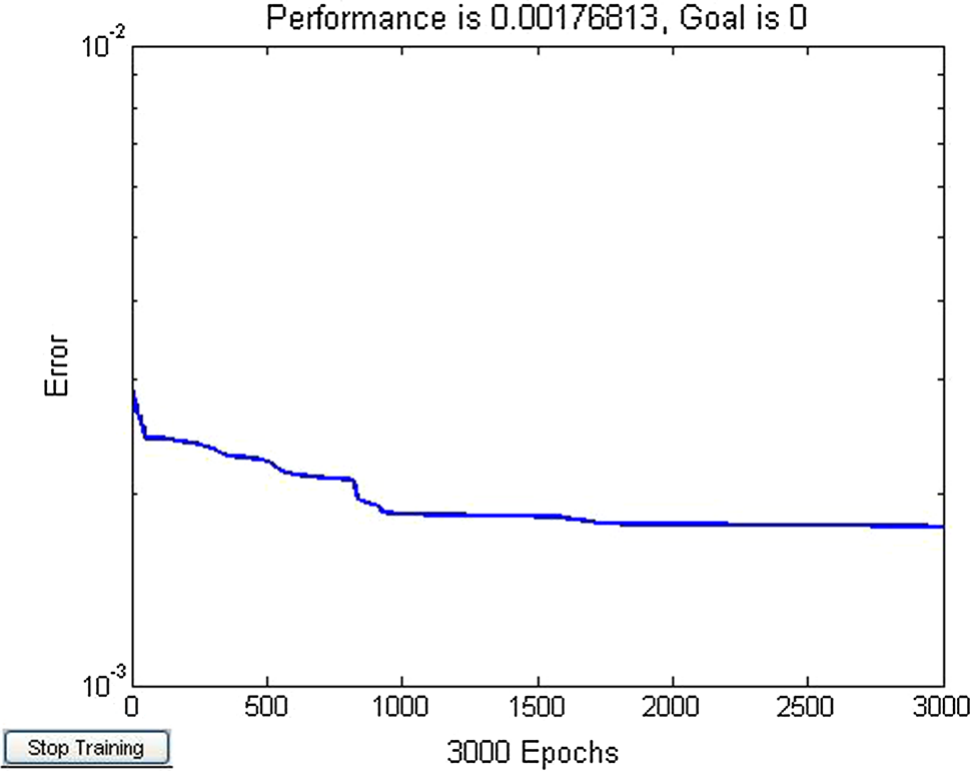
Training error curve of network A (average divide to 10)

Activation function of hidden layer units is *f* = *e* ^*x*^. After adjustment, the hidden layer neurons *s* = 45. Use Levenberg–Marquardt training algorithm. Training integrand network A 3000 steps, error convergence curve is shown in Fig. 5.

Network A training sample set of each vertex in a hypercube is calculated and simulated with network B, and the sample set is shown in Table 5. The results are substituted to Eq. (7) to get the result of reliability.

**Table 5:** Simulative sample set of network B in example 2

*P*	0	0	0	0	8	8	8	8
*E*	0	0	4	4	0	0	4	4
*I*	0.2	1.8	0.2	1.8	0.2	1.8	0.2	1.8

**Fig. 6:**
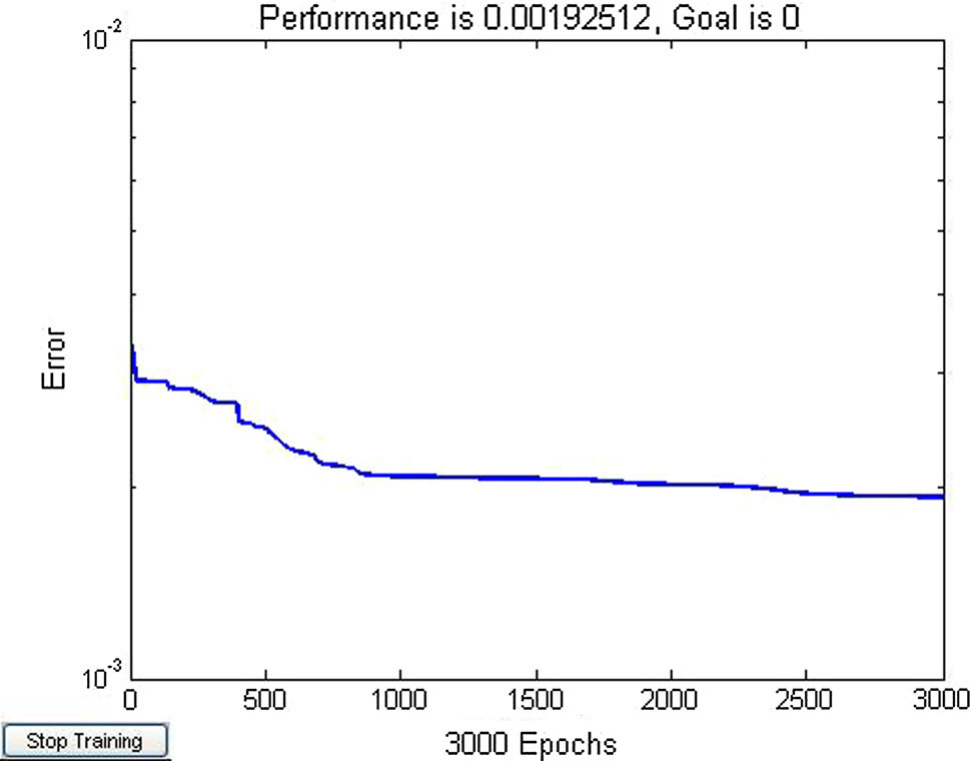
Training error curve of network A (average divide to 20)

In order to observe the training sample quantity influence on computational accuracy, variables *P*, *E*, *I* are, respectively, divided into 20 parts in the range of [*μ*_*P*_ − 4*σ*_*P*_, *μ*_*P*_ + 4*σ*_*P*_], [*μ*_*E*_ − 4*σ*_*E*_, *μ*_*E*_ + 4*σ*_*E*_] and [*μ*_*I*_ − 4*σ*_*I*_, *μ*_*I*_ + 4*σ*_*I*_]. Use the same method to construct and calculate the input and output sample points. Training integrand network A after 3000 steps, error convergence curve is shown in Fig. 6.

Use network B to simulate and calculate sample set in Table 5; then, we get the structure reliability. In order to demonstrate the efficiency and accuracy of the proposed method, reliability calculated using the mean value first-order second moment (MVFOSM) method, Hasofer–Lind (HL) method and Monte Carlo simulation (MCS) method is also given in Table 6. All methods compared with MCS with 1,000,000 samples are shown in Table 6. The result calculated using MCS is viewed as “true value” for the comparisons.

**Table 6:** Example 2 reliability calculation results

Method	The proposed method (divided into 10 parts)	The proposed method (divided into 20 parts)	MVFOSM	HL	MCS
Reliability	0.9696	0.9845	0.9956	0.9996	0.9993
Relative error (%)	2.972	1.481	0.37	0.03	0

Figures 5 and 6 show that under the condition of same training steps, the training sample points in Fig. 6 (divided into 20 parts) are more than in Fig. 5 (divided into 10 parts). The network error in Fig. 6 is slightly bigger than in Fig. 5. At the same time, more training sample points are beneficial to approximate integrand surface, so the training result of each variable divided into 20 parts is better than 10 parts. That is, the more training sample points of integrand, the accuracy of original function is more improved and the reliability calculation accuracy is also higher.

From Table 6, compared with the example 1, the accuracy of example 2 is affected by the number of sample points decrease, the computational accuracy slightly decrease. But with the increase in the number of sample points, computational accuracy improves. When sample points of each variable doubled, the relative error reduced from 2.972 % to 1.481 %. It can be seen that the results calculated using the proposed method are accurate when compared to MCS. However, the efficiency of the proposed method is higher than MCS because it requires fewer samples. The neural networks are trained using 3000 steps to meet the requirement.

## Conclusions

This paper presents a reliability analysis method based on dual neural network. This method has higher accuracy than traditional first-order second moment method (the mean value first-order second moment (MVFOSM) method, Hasofer–Lind (HL) method). When the training sample points are so few (each variable divided into 10 parts), the computational accuracy of this method is <3 %. It still meets the precision requirement in engineering. With the increase in sample points (each variable divided into 20 parts), computational accuracy also increases accordingly.

The major contribution of this paper is that a dual neural network is developed which can be used for integral and multiple integral efficiently. Because of the training sample set come from the integrand, we do not need to establish the original function and mapping relationship of the random variables. Thus, we select sample set conveniently. It will improve the computational accuracy of reliability. In addition, this paper adopted uniform sampling method to select the training sample points set in the variable space, to a certain extent, which affect the calculation accuracy and efficiency. In the later work, uniform sampling method will combine with orthogonal experiment design method [33], super Latin cube sampling method [34] to select training sample points set. It is expected to further enhance accuracy and efficiency of the algorithm in this paper.

Modeled on the structure principle of the dual neural network, the proposed method in this paper can easily expand to solve problems of partial differential equation and fractional-order derivative equation. This will provide a new thought, which can solve elastic mechanics, structural dynamics, viscoelastic mechanics analysis in large complex structure. At the same time, the proposed method is also expected to reveal how to utilize the existing mathematics (mechanical) model to determine network structure of the neural network. At this moment, neural network not only is a black box system that depends on the input and output data, but also can make existing knowledge and rule (equations) effectively integrate into the network. The structure of neural network is more reasonable and rational; this rational neural network calculation method plays a bigger role in the problems of structure analysis.
